# Machine Learning to Tailor Intermittent Fasting for Blood Pressure Improvement

**DOI:** 10.3390/nu18040667

**Published:** 2026-02-18

**Authors:** Shula Shazman

**Affiliations:** Department of Mathematics and Computer Science, The Open University of Israel, Ra’anana 4353701, Israel; shulash@openu.ac.il

**Keywords:** intermittent fasting, machine learning, blood pressure, precision medicine, decision trees

## Abstract

**Background/Objectives:** Intermittent fasting (IF) has shown feature effectiveness in reducing blood pressure, highlighting the need for personalized intervention strategies. **Methods:** To address this, a machine learning framework was developed to predict the likelihood of blood pressure improvement (≥5 mmHg systolic reduction) across different IF and calorie restriction protocols in premenopausal women without diagnosed hypertension. **Results:** The model achieved 77% accuracy and an AUC of 0.8 in distinguishing responders from non-responders. Logistic regression analysis identified dietary intervention type as the strongest predictor of success, with Intermittent Energy and Carbohydrate Restriction + free Protein and Fat (IECR + FF) and Intermittent Energy and Carbohydrate Restriction + free Protein and Fat (IECR) protocols showing the highest effectiveness (coefficients 0.55 and 0.41 respectively). Decision tree analysis revealed age in years as a critical stratification factor, with younger patients (≤47 years) responding optimally to IECR + FF combinations, while older patients benefited from IECR, Continuous Energy Restriction (CER), or Intermittent Energy Restriction (IER) approaches. Notably, waist-to-hip ratio emerged as the strongest negative predictor, indicating that central adiposity significantly impedes blood pressure improvement regardless of intervention type. Higher baseline HDL positively predicted success, while elevated LDL and the DER diet were associated with poor outcomes. The complementary analytical approaches demonstrated that logistic regression and decision tree methods highlight different aspects of the data, with the former identifying independent linear associations and the latter suggesting potential non-linear interactions and candidate thresholds involving age years, dietary intervention type, baseline blood pressure, and metabolic markers. **Conclusions:** This exploratory, hypothesis-generating analysis was conducted in a cohort of premenopausal women without diagnosed hypertension and is not intended to inform clinical decision-making. The observed patterns should be interpreted as preliminary and may reflect sample-specific effects or model instability. Confirmation in larger, independent, and more diverse populations is essential before any clinical relevance can be inferred.

## 1. Introduction

Hypertension affects over 1.13 billion people worldwide and remains the leading modifiable risk factor for cardiovascular disease, stroke, and premature mortality [1,2]. Despite the availability of effective pharmacological treatments, many patients experience suboptimal blood pressure control, medication intolerance, or prefer lifestyle-based interventions as first-line therapy [3,4]. This clinical reality has intensified interest in evidence-based dietary approaches that can provide meaningful blood pressure reductions while addressing the growing preference for non-pharmacological interventions.

Intermittent fasting (IF) has emerged as a promising therapeutic strategy for blood pressure management in various populations, encompassing various protocols including Daily Energy Restriction (DER), Intermittent Energy and Carbohydrate Restriction (IECR), Continuous Energy Restriction (CER), Intermittent Energy Restriction (IER), and combined approaches [5,6]. The scientific foundation for IF extends beyond simple caloric reduction, with emerging evidence suggesting distinct metabolic pathways activated during fasting periods that may independently contribute to cardiovascular benefits.

Meta-analyses have demonstrated that IF interventions can achieve clinically significant blood pressure reductions, with systolic blood pressure decreases ranging from 2–8 mmHg across different populations [7,8]. A comprehensive umbrella review of randomized controlled trials revealed consistent benefits across multiple IF protocols, though effect sizes varied considerably based on intervention duration, baseline characteristics, and adherence patterns [8]. Recent network meta-analyses have shown that modified alternate-day fasting may be particularly effective, achieving systolic blood pressure reductions of approximately 7 mmHg [9]. Time-restricted eating protocols, which limit food intake to specific hours within a 24-h period, have shown particular promise in reducing both systolic and diastolic blood pressure even in the absence of significant weight loss, suggesting weight-independent mechanisms of action [10,11,12].

The proposed mechanisms underlying IF’s blood pressure effects are multifaceted and interconnected. Weight loss, when it occurs, contributes significantly to blood pressure reduction, with studies demonstrating approximately 1 mmHg decrease in systolic blood pressure per kilogram of weight lost [13]. However, research has increasingly identified weight-independent pathways, including improved insulin sensitivity and glucose regulation, which appear to enhance endothelial function and reduce arterial stiffness. Ketone body production during fasting periods, particularly beta-hydroxybutyrate, has been associated with reduced inflammation through inhibition of the NLRP3 inflammasome and decreased oxidative stress, both of which are implicated in hypertension pathogenesis [14,15]. Additionally, favorable changes in the renin–angiotensin–aldosterone system, sympathetic nervous system activity modulation, and alterations in gut microbiome composition have been documented in IF interventions [9,10,16,17].

Recent mechanistic studies have revealed that IF may enhance autophagy in smooth vascular muscle cells and endothelial progenitor cells, improve nitric oxide bioavailability, and reduce endothelial dysfunction markers [18,19]. The circadian rhythm realignment achieved through time-restricted feeding may also contribute to blood pressure improvements by optimizing the natural nocturnal blood pressure dipping pattern, which is often disrupted in stage 1 hypertension (HTN) individuals [20]. Furthermore, emerging evidence suggests that IF may beneficially modulate adipokine secretion, particularly reducing leptin and increasing adiponectin levels, which influence blood pressure regulation through multiple pathways [21].

However, clinical implementation of IF for blood pressure management faces a critical challenge: substantial inter-individual variability in treatment response. While some patients achieve remarkable blood pressure improvements, others show minimal or no benefit from the same intervention protocol [16,17]. Population-level studies have documented response rates ranging from 40% to 70%, depending on the IF protocol and study population, indicating that a significant proportion of individuals may not benefit from a given approach [7,8,9].

This heterogeneity in response suggests that patient-specific factors—including baseline metabolic profile, anthropometric characteristics, and cardiovascular risk status—may determine IF efficacy. Recent investigations have identified several potential moderators of IF response, including baseline insulin resistance, inflammatory markers, age in years, sex, menopausal status in women, medication use, sleep patterns, and physical activity levels [8,9,12,22]. Some studies suggest that individuals with higher baseline metabolic dysfunction may experience greater benefits, while others indicate that certain IF protocols may be more effective in metabolically healthy individuals seeking blood pressure optimization [13,14,23].

Current clinical practice relies primarily on trial-and-error approaches to IF protocol selection, potentially leading to suboptimal outcomes and reduced patient adherence, and prolonged time to effective blood pressure control. The lack of evidence-based guidance for matching patients to specific IF protocols represent a significant gap in translating research findings into clinical practice. Moreover, the dropout rates in IF interventions, ranging from 10% to 40% across studies, underscore the importance of selecting appropriate protocols that align with individual preferences, lifestyles, and physiological characteristics [15,18,24,25].

The emergence of precision medicine approaches in cardiovascular care has highlighted the potential for individualized treatment selection based on patient phenotyping [26,27]. Traditional cardiovascular risk stratification tools, while valuable for assessing overall risk, have limited utility in predicting response to specific lifestyle interventions. The complexity of factors influencing IF efficacy—involving interactions between metabolic, anthropometric, behavioral, and potentially genetic determinants—exceeds the capacity of conventional statistical approaches and clinical intuition. Machine learning techniques, particularly those that maintain clinical interpretability, offer promising tools for identifying patient subgroups most likely to benefit from specific interventions [28,29]. Unlike traditional regression models that assume linear relationships and require predetermined interactions, machine learning algorithms can identify complex, nonlinear patterns and interactions across multiple features. Several cardiovascular applications have demonstrated the superiority of machine learning approaches in risk prediction, treatment response prediction, and patient stratification [19,20,28,29,30].

Decision tree algorithms create hierarchical classification models through sequential binary splits based on predictor features, where each split identifies an optimal threshold that maximizes separation between outcome classes. They excel at capturing non-linear relationships and complex interactions without requiring assumptions about data distribution, and their visual structure naturally reveals which features are most important and at what thresholds they become clinically relevant. However, standard decision trees may oversimplify relationships at terminal nodes, potentially missing subtle associations between predictors and outcomes.

To address these complementary strengths and limitations, this study employed both standard decision tree analysis and logistic regression modeling. Decision trees provide transparent identification of critical thresholds and interaction patterns—revealing, for example, specific age in years cutoffs or metabolic values that determine treatment selection—while logistic regression quantifies the independent linear effects of each predictor and the magnitude of each factor’s contribution to treatment success. Additionally, Logistic Model Tree (LMT) classifiers were implemented to combine these approaches within a unified framework, embedding logistic regression models at terminal nodes to capture subtle relationships that simple classifications might miss [31]. This multi-method strategy enables comprehensive understanding: decision trees identify patient subgroups and decision rules for clinical stratification, logistic models reveal the direction and strength of individual predictor effects, and LMT provides an integrated hierarchical structure that mirrors clinical decision-making processes, where broad categorizations are followed by increasingly nuanced assessments.

Recent applications of machine learning in nutritional epidemiology and precision nutrition have demonstrated the potential for data-driven approaches to identify responders to dietary interventions. Studies examining response to Mediterranean diet, low-carbohydrate diets, and plant-based interventions have successfully employed machine learning techniques to develop predictive models with clinically meaningful accuracy [7,8,9,21,22,32]. However, these approaches remain underutilized in IF research, despite the clear need for personalized protocol selection.

Despite the growing evidence base for IF in blood pressure management, no studies have systematically applied machine learning approaches to personalize IF protocol selection. The identification of predictive biomarkers and clinical characteristics that can guide intervention choice remains an unmet clinical need. Furthermore, the optimal approach to patient stratification for IF interventions—whether based on baseline blood pressure, metabolic parameters, or demographic factors—has not been established.

Several critical questions remain unanswered: Do patients of different ages require different dietary intervention approaches? What is the relative importance of body composition (particularly central adiposity) versus metabolic markers in predicting IF success? Do specific IF protocols show differential effectiveness across age groups and metabolic phenotypes? Can we identify which baseline patient characteristics—such as age in years, waist-to-hip ratio, HDL, and LDL levels—most strongly predict treatment response to guide personalized protocol selection?

Therefore, this study aimed to develop and validate a machine learning framework for personalizing intermittent fasting interventions in blood pressure management in premenopausal women. Specifically, this study sought to: (1) identify the key clinical, metabolic, and anthropometric determinants of blood pressure improvement with IF; (2) develop an interpretable predictive model using Logistic Model Tree methodology; (3) determine whether age-based stratification and body composition parameters, particularly central adiposity measured by waist-to-hip ratio, can identify patients most likely to benefit from specific IF protocols; (4) evaluate the differential predictive patterns between calorie restriction and intermittent fasting protocols; and (5) a future objective is to create a clinically implementable decision framework that can guide personalized IF protocol selection in practice.

## 2. Materials and Methods

### 2.1. Study Design and Reporting Standards

This study developed and internally validated a machine learning-based prediction model for identifying individuals likely to achieve blood pressure improvement through intermittent fasting interventions. The study follows the TRIPOD + AI (Transparent Reporting of a multifeature prediction model for Individual Prognosis Or Diagnosis + Artificial Intelligence) reporting guidelines for studies developing prediction models using machine learning methods [33].

#### 2.1.1. Study Objectives

The objectives of the present study were to: (1) identify key predictive features distinguishing responders from non-responders, (2) determine optimal decision thresholds for clinical stratification, and (3) compare predictive patterns between intermittent fasting and continuous calorie restriction protocols. These findings lay the groundwork for the future development of a clinical decision support model that predicts the probability of blood pressure improvement (defined as reduction in pulse pressure as first use) following 12 weeks of intermittent fasting or calorie restriction intervention.

#### 2.1.2. Target Population and Intended Use

The model is intended for use in adult women (aged 18–65 years) who are overweight or obese (BMI ≥ 25 kg/m^2^) and considering intermittent fasting interventions for blood pressure management. The model applies to individuals without diagnosed cardiovascular disease or diabetes at baseline, who are not currently taking antihypertensive medications, and who can adhere to dietary interventions. The clinical setting is primary care or nutrition counseling contexts where personalized intervention recommendations are needed.

### 2.2. Data Source and Study Population

#### 2.2.1. Source Studies

Individual-level data were obtained from two published randomized controlled trials conducted by Harvie et al. [34,35], which examined the effects of various intermittent fasting and calorie restriction protocols on metabolic and anthropometric parameters. Both studies were conducted in the United Kingdom and recruited participants through public advertisements and primary care referrals.

Study 1 (Harvie et al. 2011) [34]:

- Design: Randomized controlled trial comparing Intermittent Energy Restriction (IER) versus Continuous Energy Restriction (CER);
- Duration: 6 months intervention with measurements at baseline and 12 weeks;
- Setting: Community-based recruitment in South Manchester, UK;
- Recruitment period: 2006–2008;
- Sample size: 107 premenopausal women;
- Inclusion criteria: Age 30–45 years, BMI 24–40 kg/m^2^, family history of breast cancer;
- Exclusion criteria: Diabetes, cardiovascular disease, current pregnancy or breastfeeding, use of medications affecting metabolism.

Study 2 (Harvie et al. 2013) [35]:

- Design: Randomized controlled trial comparing Intermittent Energy and Carbohydrate Restriction (IECR), IECR with free protein and fat (IECR + PF), versus Daily Energy Restriction (DER);
- Duration: 3 months intervention with measurements at baseline and 12 weeks;
- Setting: Community-based recruitment in South Manchester, UK;
- Recruitment period: 2009–2011;
- Sample size: 115 overweight or obese women;
- Inclusion criteria: Age 30–45 years, BMI ≥ 25 kg/m^2^;
- Exclusion criteria: Similar to Study 1.

#### 2.2.2. Participant Characteristics

The combined dataset included 222 premenopausal women with the following baseline characteristics:

- Age: Mean 44.0 years (SD 7.0, range 30–45);
- BMI: Mean 31.0 kg/m^2^ (SD 5.0, range 24–40);
- Baseline systolic blood pressure: Mean 121.0 mmHg (SD 17.0);
- Baseline diastolic blood pressure: Mean 75.0 mmHg (SD 11.0).

All participants were free of diagnosed cardiovascular disease and diabetes at enrollment. Table 1 presents the mean and standard deviation of all collected features. As shown in the table, the data for each individual included the following features: age in years type of diet, weight, hip circumference, waist circumference, waist-to-hip ratio, Body Mass Index (BMI), systolic blood pressure (S-BP), diastolic blood pressure (D-BP), total cholesterol, Low-Density Lipoprotein (LDL), High-Density Lipoprotein (HDL), fasting glucose, and triglycerides.

Except for age in years and diet type, all features had two recorded values: baseline measurement and a value after 12 weeks of intervention. In this study, three combination methods were used: Mean Arterial Pressure (MAP), which offers a clinically relevant measure by accounting for the longer duration of diastole; Pulse Pressure, which is calculated as systolic pressure minus diastolic pressure; and a weighted sum of systolic and diastolic pressures, with age-based adjustments to the weights. Specifically, for individuals aged under 50 years, equal weights were assigned (0.5 for both systolic and diastolic); for those aged 50 to 65 years, the weights were 0.65 for systolic and 0.35 for diastolic; and for individuals over 65, the weights were 0.75 for systolic and 0.25 for diastolic.

#### 2.2.3. Interventions

Table 2 provides details of each intervention protocol. The interventions were grouped into two categories:

**Intermittent Fasting (IF) Protocols (*n* = 147):**

- IER (Intermittent Energy Restriction): Daily restriction to approximately 650 kcal for 2 days per week, with Mediterranean-style diet on remaining 5 days;
- IECR (Intermittent Energy and Carbohydrate Restriction): Daily restriction to approximately 650 kcal with carbohydrate limitation to <50 g for 2 days per week, with carbohydrate restriction (<50 g) on restriction days;
- IECR + PF (IECR with free Protein and Fat): 2 days per week with unrestricted protein and fat intake but severe carbohydrate restriction (<50 g), approximately 1000 kcal/day on restriction days.

**Continuous Calorie Restriction (CR) Protocols (*n* = 75):**

- CER (Continuous Energy Restriction): Daily energy restriction of approximately 1500 kcal/day, 7 days per week, Mediterranean-style diet;
- DER (Daily Energy Restriction): Daily energy restriction of approximately 1400 kcal/day, 7 days per week, standard healthy eating guidelines.

All interventions included monthly group support sessions with dietitians and were supervised throughout the 12-week period analyzed in this study.

#### 2.2.4. Data Availability

Individual-level data used in this analysis were obtained with permission from the original study investigators. The dataset is publicly available at: https://github.com/shulash/Blood-Pressure-and-Intermittent-Fasting/issues/1 (accessed on 12 January 2026). As this study constitutes a secondary analysis of de-identified data from previously published trials, additional ethical approval was not required.

### 2.3. Outcome Definition

#### 2.3.1. Primary Outcome

The primary outcome was clinically significant systolic blood pressure improvement, defined as a reduction of ≥5 mmHg between the baseline and 12-week measurements. This threshold was selected to address both clinical significance and methodological rigor, representing a reduction associated with meaningful cardiovascular benefit while providing robust model discrimination.

Primary Outcome Classification:

Improved (TRUE): SBP reduction ≥ 5 mmHg from baseline to 12 weeks.

Not Improved (FALSE): SBP reduction < 5 mmHg from baseline to 12 weeks.

#### 2.3.2. Sensitivity Analyses

To evaluate model stability across different outcome definitions and demonstrate that findings were not artifacts of threshold selection, eight additional outcome measures were analyzed:

1. SBP ≥ 5 mmHg—Primary threshold representing clinically meaningful systolic blood pressure reduction, used to identify responders with significant cardiovascular benefit.
2. SBP ≥ 3 mmHg—Conservative threshold capturing modest improvements, testing whether predictive patterns hold with more lenient criteria.
3. SBP > 0 mmHg—Any improvement threshold demonstrating the impact of threshold choice on model performance and identifying the maximal pool of potential responders.
4. DBP ≥ 3 mmHg—Alternative blood pressure component examining whether diastolic reduction follows similar predictive patterns and whether different predictors emerge for DBP-specific response.
5. SBP ≥ 5 mmHg AND DBP ≥ 3 mmHg—Composite criterion requiring improvement in both blood pressure components, representing the most stringent definition of treatment response.
6. SBP ≥ 5 mmHg OR DBP ≥ 3 mmHg—Composite criterion capturing patients who achieve clinically meaningful reduction in either component.
7. Pulse Pressure > 0 mmHg—Original analytical approach (PP = SBP − DBP) maintained for comparison, representing any reduction in pulse pressure as a marker of arterial compliance improvement.
8. Pulse Pressure ≥ 3 mmHg—Pulse pressure with clinically meaningful threshold, providing comparison with composite measures while focusing on arterial stiffness. Testing whether predictive patterns hold with more lenient criteria.
9. Pulse Pressure ≥ 5 mmHg—Pulse pressure with clinically meaningful threshold, providing comparison with composite measures while focusing on arterial stiffness.

#### 2.3.3. Rationale for Multi-Outcome Approach

This comprehensive analytical strategy serves several critical purposes:

Addressing Model Stability Concerns:

By demonstrating consistent predictive patterns across multiple outcome definitions with varying stringency, this approach evaluates whether the machine learning models identify robust associations or are overfit to specific threshold choices. Consistency across outcomes strengthens confidence in the identified predictors and decision rules.

Clinical Applicability:

Different thresholds reflect different clinical scenarios:

- The ≥5 mmHg threshold aligns with guideline-recommended targets and meta-analytic effect sizes.
- The ≥3 mmHg threshold represents conservative improvement that may still confer benefit.
- The composite criteria (AND/OR) reflect real-world scenarios where patients may show preferential SBP or DBP response.
- Alternative component analysis (DBP, pulse pressure) addresses the possibility that different IF protocols may affect blood pressure components differentially.

Transparency and Reproducibility:

Presenting multiple outcome definitions with their respective model performance metrics provides readers with complete information to assess whether conclusions depend on arbitrary analytical choices or represent genuine patterns in the data.

Mechanistic Insights:

Different predictors may emerge for different types of improvement. For example, factors predicting any improvement (>0 mmHg) may differ from those predicting clinically significant reduction (≥5 mmHg), potentially revealing different mechanistic pathways or dose–response relationships.

#### 2.3.4. Primary Outcome Selection Criteria

SBP reduction ≥ 5 mmHg was selected as the primary outcome based on:

Clinical Significance: A 5 mmHg SBP reduction is associated with approximately 10% reduction in cardiovascular mortality and 7% reduction in all-cause mortality, representing a clinically meaningful treatment effect [36].

Evidence Alignment: This threshold is consistent with effect sizes reported in systematic reviews and meta-analyses of intermittent fasting interventions (range: 2–8 mmHg), representing a realistic and clinically relevant treatment goal for lifestyle modifications [37].

Guideline Consistency: Major hypertension guidelines recognize 5 mmHg as a meaningful reduction threshold for treatment evaluation and risk reclassification [38].

Predictive Performance: Among outcomes tested, SBP ≥ 5 mmHg achieved strong model discrimination (AUC = 0.79 with LMT, AUC = 0.80 with Random Forest) while maintaining balanced accuracy (70–74%), demonstrating that this threshold provides both clinical relevance and robust prediction.

Clinical Interpretability: Systolic blood pressure is the primary treatment target in contemporary hypertension management, making SBP-based outcomes more readily interpretable and actionable for clinicians compared to composite measures like pulse pressure.

While pulse pressure reduction ≥ 3 mmHg yielded marginally higher AUC (0.80) in preliminary analyses, SBP ≥ 5 mmHg was prioritized for the primary analysis due to its superior clinical applicability, direct alignment with treatment guidelines, and broader interpretability in cardiovascular risk management.

#### 2.3.5. Blood Pressure Measurement Protocol

In both source studies, blood pressure was measured according to standardized protocols:

- Measurements taken after 5 min of seated rest;
- Automated oscillometric devices used (Omron M5-I, Omron Healthcare, Kyoto, Japan);
- Three consecutive measurements taken at 1-min intervals;
- Average of the three measurements used for analysis;
- Same arm used for all measurements within each participant;
- Measurements conducted at the same time of day (±2 h) for baseline and follow-up;
- Participants instructed to avoid caffeine and exercise for 2 h prior to measurement.

### 2.4. Predictor Features

#### Candidate Predictors

A total of 13 baseline features were included as potential predictors, selected based on clinical relevance, availability in routine practice, and hypothesized biological mechanisms linking them to blood pressure response:

**Demographic Features:**

- Age (years, continuous).

**Intervention Features:**

- Diet type (categorical: CER, DER, IER, IECR, IECR + PF).

**Anthropometric Features:**

- Weight (kg, continuous);
- Waist circumference (cm, continuous);
- Hip circumference (cm, continuous);
- Waist-to-hip ratio (continuous, calculated);
- Body Mass Index (kg/m^2^, continuous, calculated).

**Cardiovascular Features:**

- Systolic blood pressure (mmHg, continuous);
- Diastolic blood pressure (mmHg, continuous).

**Metabolic Features:**

- Total cholesterol (mmol/L, continuous);
- LDL cholesterol (mmol/L, continuous);
- HDL cholesterol (mmol/L, continuous);
- Fasting glucose (mmol/L, continuous);
- Triglycerides (mmol/L, continuous).

All predictors were measured at baseline (Week 0) prior to intervention commencement. No predictor features from follow-up time points were included to ensure that the model could be applied at the point of intervention selection.

### 2.5. Sample Size and Statistical Power

#### 2.5.1. Sample Size Considerations

The dataset included 222 participants with complete data after exclusions. Following the events-per-feature (EPV) rule for prediction modeling, with a binary outcome and 13 candidate predictors, the minimum recommended sample size was 130 participants (10 events per predictor) [39].

Distribution of Primary Outcome (SBP ≥ 5 mmHg):

- Blood pressure improved: 128 participants (57.7%);
- Blood pressure not improved: 94 participants (42.3%).

This provides an EPV of approximately 7.2 for the minority class (94 events/13 predictors).

Assessment of EPV Adequacy:

The EPV ratio of 7.2 for the minority class fell within the acceptable range (5–10) though below the ideal threshold of 10 events per predictor. While the total sample size (*n* = 222) substantially exceeded the minimum recommended sample size of 130 and the outcome distribution was relatively balanced (57.7% vs. 42.3%), the EPV for the minority class suggests that model parsimony should be prioritized. Predictor inclusion was based on both statistical significance and clinical relevance to avoid overfitting, with cross-validation procedures implemented to assess model stability.

#### 2.5.2. Power for Model Performance

Based on simulation studies for similar binary classification problems [40], a sample size of 222 with the observed outcome distribution (57.7% vs. 42.3%) provides >80% power to detect a model with AUC of 0.75 versus a null model (AUC = 0.50) at α = 0.05. The relatively balanced outcome distribution enhances the reliability of model performance estimates compared to highly imbalanced datasets.

Sensitivity Analysis Considerations:

Given that seven different outcome definitions were evaluated (primary outcome: SBP ≥ 5 mmHg; sensitivity analyses: SBP ≥ 3 mmHg, SBP > 0 mmHg, DBP ≥ 3 mmHg, SBP ≥ 5 AND DBP ≥ 3, SBP ≥ 5 OR DBP ≥ 3, PP ≥ 5 mmHg, PP > 0 mmHg), outcome distributions varied across analyses. The most stringent threshold (SBP ≥ 5 AND DBP ≥ 3) resulted in the lowest proportion of responders and thus the most conservative EPV ratio, while the most lenient threshold (SBP > 0 or PP > 0) provided the highest proportion of responders. The primary outcome (SBP ≥ 5 mmHg) with its balanced distribution (57.7% vs. 42.3%) and adequate EPV (7.2) provided a robust foundation for model development. Cross-validation procedures implemented in all analyses provided additional protection against overfitting across the range of outcome definitions tested.

### 2.6. Data Preprocessing and Feature Engineering

#### 2.6.1. Data Cleaning

Data Type Verification: All continuous features were verified to be numeric and within biologically plausible ranges. Categorical features (diet type) were encoded appropriately with no invalid categories detected.

#### 2.6.2. Feature Transformations

No transformations were applied to predictor features to maintain model interpretability and clinical applicability. Logistic Model Tree classifiers can handle non-linear relationships without requiring predictor transformations.

Derived Features:

- BMI calculated as: Weight (kg)/[Height (m)]^2^;
- Waist-to-hip ratio calculated as: Waist circumference (cm)/Hip circumference (cm).

#### 2.6.3. Standardization

Continuous predictor features were not standardized prior to analysis for the following reasons:

1. Decision tree-based algorithms are invariant to monotonic transformations;
2. Maintaining original units enhances clinical interpretability;
3. Logistic regression components within LMT can handle features on different scales.

### 2.7. Model Development

#### 2.7.1. Algorithm Selection

Three tree-based classification algorithms were evaluated using Weka software (version 3.8.6) [41]:

1. J48 Decision Tree:

- Weka implementation of the C4.5 algorithm;
- Creates interpretable rule-based decision trees;
- Uses information gain ratio for split selection;
- Includes post-pruning to prevent overfitting.

2. Logistic Model Tree (LMT):

- Hybrid algorithm combining decision trees with logistic regression;
- Builds classification tree with logistic regression models at leaf nodes;
- Uses LogitBoost algorithm for model fitting;
- Provides both hierarchical decision structure and probabilistic predictions.

3. Random Forest:

- Ensemble method creating multiple decision trees;
- Uses bootstrap aggregating (bagging) for tree diversity;
- Predictions made by majority voting across trees;
- Reduces overfitting compared to single decision trees.

#### 2.7.2. Hyperparameter Settings

J48 Decision Tree Parameters:

- Confidence factor for pruning: 0.25 (default);
- Minimum number of instances per leaf: 2 (default);
- Binary splits: True;
- Subtree raising: True;
- Reduced error pruning: False.

Logistic Model Tree Parameters:

- Fast regression: True (uses simple regression instead of LogitBoost);
- Minimum number of instances: 15;
- Number of boosting iterations: −1 (automatic determination);
- Weight trimming beta: 0 (no weight trimming);
- Error on probabilities: False;
- Use AIC for model selection: False.

Random Forest Parameters:

- Number of trees: 100;
- Number of features to consider at each split: √p (square root of total predictors ≈ 4);
- Maximum depth: Unlimited;
- Minimum samples per leaf: 1;
- Bootstrap samples: True;
- Out-of-bag error estimation: True.

#### 2.7.3. Treatment of Class Imbalance

The outcome distribution was relatively balanced (70% improved vs. 30% not improved), falling within an acceptable range where no special class balancing techniques were strictly required. Although the distribution showed some imbalance with approximately a 2.4:1 ratio, this level of imbalance is generally not considered severe enough to warrant resampling techniques, which can introduce bias or reduce generalizability. No resampling (oversampling, undersampling), class weighting adjustments, or synthetic minority oversampling technique (SMOTE) were applied. The decision tree-based algorithms used (J48, LMT, Random Forest) were relatively robust to moderate class imbalance, and the cross-validation approach provided reliable performance estimates despite the outcome distribution.

### 2.8. Model Validation and Performance Assessment

#### 2.8.1. Internal Validation Strategy

10-Fold Cross-Validation: The 222-participant dataset was randomly partitioned into 10 approximately equal-sized subsets (folds), stratified by outcome to maintain similar outcome proportions across folds. The model was trained in 9 folds (approximately 200 participants) and tested on the remaining fold (approximately 22 participants). This process was repeated 10 times with each fold serving as the test set once. Performance metrics were averaged across all 10 folds. Rationale: 10-fold cross-validation was selected as it provides a good balance between bias and variance in performance estimates, uses data efficiently (90% for training, 10% for testing), and is a widely accepted standard for datasets of this size [42].

#### 2.8.2. Performance Metrics

Primary Performance Metrics:

1. Area Under the ROC Curve (AUC):

- Measures discrimination ability across all possible classification thresholds;
- Interpretation: Probability that a randomly selected participant with BP improvement has a higher predicted probability than a randomly selected participant without improvement;
- Range: 0.5 (no discrimination) to 1.0 (perfect discrimination);
- Clinical interpretation: AUC 0.70–0.80 considered acceptable; >0.80 excellent.

2. Classification Accuracy:

- Proportion of correctly classified participants;
- Calculated as: (True Positives + True Negatives)/Total;
- Default classification threshold: 0.5 probability.

Secondary Performance Metrics:

3. Sensitivity (Recall):

- Proportion of true responders correctly identified;
- Calculated as: True Positives/(True Positives + False Negatives).

4. Specificity:

- Proportion of true non-responders correctly identified;
- Calculated as: True Negatives/(True Negatives + False Positives).

5. Positive Predictive Value (Precision):

- Proportion of predicted responders who truly respond;
- Calculated as: True Positives/(True Positives + False Positives).

6. Negative Predictive Value:

- Proportion of predicted non-responders who truly do not respond;
- Calculated as: True Negatives/(True Negatives + False Negatives).

7. F1 Score:

- Harmonic mean of precision and recall;
- Calculated as: 2 × (Precision × Recall)/(Precision + Recall).

### 2.9. Model Interpretation and Clinical Translation

#### 2.9.1. Decision Tree Visualization

The selected Logistic Model Tree structure was visualized to illustrate:

- Root split criteria (primary stratification feature);
- Secondary branch points;
- Terminal node classifications;
- Path from root to each leaf node representing clinical decision pathways.

#### 2.9.2. Feature Importance

Feature importance was assessed through:

- Position in tree hierarchy (features used for early splits are more important).

#### 2.9.3. Clinical Subgroup Identification

The tree structure naturally stratifies patients into clinical subgroups based on:

- Baseline blood pressure category (Normal BP vs. Stage 1 hypertension (HTN));
- Intervention type (IF vs. CR);
- Metabolic vs. anthropometric feature profiles.

## 3. Results

Three machine learning algorithms (J48 decision tree, Logistic Model Tree [LMT], and Random Forest) were evaluated across nine different blood pressure outcome definitions to identify the optimal combination of outcome measure, classification threshold, and modeling approach. Performance metrics across all combinations are presented in Table 3.

### 3.1. Overall Algorithm Performance

The Logistic Model Tree (LMT) classifier demonstrated the most consistent performance across outcome definitions, achieving AUC values ranging from 0.73 to 0.80 and accuracy ranging from 70% to 77%. Random Forest showed competitive performance for specific outcomes, particularly achieving the highest AUC (0.80) for the primary outcome of SBP ≥ 5 mmHg with 74% accuracy. The J48 decision tree classifier generally showed lower performance, with AUC values ranging from 0.52 to 0.74 and accuracy from 62% to 74%, though it provided the most interpretable decision rules.

#### 3.1.1. Primary Outcome Performance (SBP ≥ 5 mmHg)

For the primary outcome of clinically significant systolic blood pressure reduction (≥5 mmHg), all three classifiers achieved comparable discrimination (Table 3):

J48: Accuracy = 69%, AUC = 0.68;

LMT: Accuracy = 70%, AUC = 0.79;

Random Forest: Accuracy = 74%, AUC = 0.80.

The Random Forest and LMT classifiers both achieved strong discrimination (AUC = 0.80 and 0.79, respectively), with Random Forest showing marginally higher accuracy (74% vs. 70%). These performance metrics indicate that the models successfully distinguished between patients likely to achieve clinically meaningful blood pressure improvement and those unlikely to benefit from dietary interventions.

#### 3.1.2. Sensitivity Analysis Results

Across the nine sensitivity analyses, several patterns emerged (Table 3):

1. Pulse Pressure Outcomes:

Pulse pressure reduction ≥ 3 mmHg with LMT achieved the highest overall AUC (0.80) with 76% accuracy, suggesting that this composite measure captures arterial compliance improvements that are predictable from baseline characteristics. However, pulse pressure > 0 mmHg (any improvement) showed lower discrimination (J48 AUC = 0.65, LMT AUC = 0.76), indicating that predicting any degree of pulse pressure change is more challenging than predicting clinically meaningful reductions.

2. Systolic Blood Pressure Thresholds:

Model performance varied systematically with threshold stringency. The most lenient threshold (SBP > 0 mmHg) yielded high accuracy (77% with LMT) but moderate AUC (0.78), reflecting the ease of predicting any improvement but less discrimination between true responders and marginal changes. The intermediate threshold (SBP ≥ 3 mmHg) achieved balanced performance (LMT: 73% accuracy, AUC = 0.78), while the clinically meaningful threshold (SBP ≥ 5 mmHg) maintained strong discrimination (AUC = 0.79–0.80) with acceptable accuracy (70–74%).

3. Diastolic Blood Pressure:

DBP ≥ 3 mmHg showed moderate performance (LMT: 73% accuracy, AUC = 0.73), suggesting that diastolic improvement is somewhat less predictable from baseline characteristics than systolic improvement.

4. Composite Criteria:

Both composite outcomes (SBP ≥ 5 AND DBP ≥ 3; SBP ≥ 5 OR DBP ≥ 3) achieved identical performance with LMT (73% accuracy, AUC = 0.74) and Random Forest (73% accuracy, AUC = 0.76), indicating that requiring simultaneous improvement in both components or allowing improvement in either component yields a similar predictive accuracy.

### 3.2. Decision Tree Analysis: Key Predictive Features and Thresholds

**Figure 1:**
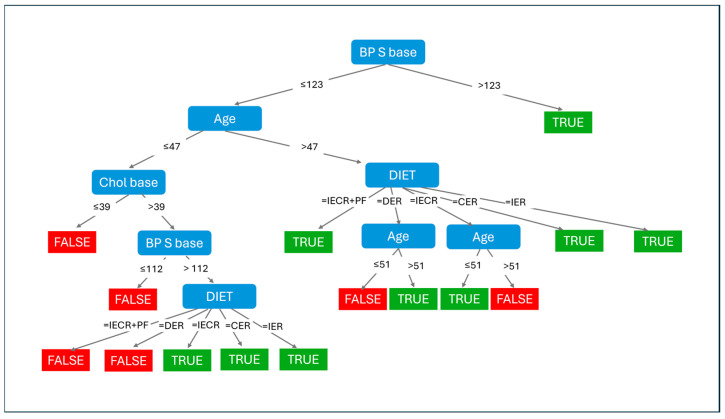
**Decision Tree for Predicting Systolic Blood Pressure Improvement (≥5 mmHg).** J48 decision tree classifier showing hierarchical decision rules based on baseline systolic blood pressure (BP S base), age, dietary intervention type (DIET), and metabolic markers. Terminal nodes show classification (TRUE = improvement, green; FALSE = no improvement, red) with sample sizes and misclassification counts in parentheses (total/misclassified).

To identify the key predictive features and their threshold values for blood pressure improvement, the J48 decision tree classifier was examined. The hierarchical structure of the decision tree is shown in Figure 1.

The decision tree structure contained 21 terminal nodes with highly feature sample sizes. Many terminal nodes contained ≤5 samples, substantially limiting the reliability of predictions from these nodes. Thresholds appearing deeper branches should be interpreted with caution, as these may represent sample-specific patterns rather than generalizable relationships. External validation is essential to determine whether these splits represent true biological transitions or statistical artifacts.

#### 3.2.1. Primary Decision Node (BP S Base ≤ 123 vs. >123 mmHg)

The tree structure revealed age-based stratification (threshold: 47 years) and diet-specific response patterns, with baseline BP S base ≤ 123 mmHg as the primary decision point. Patients with baseline SBP > 123 mmHg showed a higher probability of achieving a ≥5 mmHg reduction, with subsequent classification depending primarily on age and diet type (Figure 1). This finding indicates that individuals starting with higher baseline blood pressure have greater potential for absolute reduction, consistent with regression to the mean and ceiling effects in normal BP individuals.

#### 3.2.2. Age-Based Stratification

Age emerged as a critical secondary predictor, with distinct decision pathways for younger (≤47 years) and older (>47 years) participants:

Younger Participants (Age ≤ 47):

Among the younger individuals with baseline SBP ≤ 123 mmHg, further stratification occurred based on baseline cholesterol (threshold: 3.9 mmol/L) (Figure 1). Those with cholesterol ≤ 3.9 showed uniformly poor response (FALSE, 11.0), suggesting that very low baseline cholesterol may indicate limited metabolic responsiveness or different physiological profiles. For those with cholesterol > 3.9, an additional BP baseline threshold of 112 mmHg determined outcomes, with complex diet-specific patterns emerging at lower blood pressure levels.

Older Participants (Age > 47):

For older individuals, dietary intervention type became the dominant predictor (Figure 1). Those assigned to IECR, CER, or IER diets showed favorable outcomes (TRUE, 0.0), while those on DER diets required additional assessment based on triglyceride baseline, HDL baseline, and BPD baseline to determine response probability. This age–diet interaction pattern, if validated in independent cohorts, could potentially inform age-stratified dietary recommendations.

#### 3.2.3. Metabolic Markers in Terminal Nodes

At deeper levels of the tree, metabolic markers refined predictions within specific subgroups (Figure 1):

BMI baseline (threshold: 39.1 kg/m^2^) separated responders in the low-cholesterol, lower-blood-pressure pathway, with extreme obesity (BMI > 48.1) predicting poor outcomes.

Triglyceride baseline (threshold: 2.3 mmol/L) distinguished responders among older participants on DER diets.

HDL baseline (threshold: 1.4 mmol/L) and BPD baseline (threshold: 88 mmHg) provided final classification in specific branches.

#### 3.2.4. Terminal Node Classification

The tree identified multiple pathways to successful blood pressure improvement (TRUE outcomes-colored green) and failure to improve (FALSE outcomes-colored red), with sample sizes and error rates indicated at each terminal node (Figure 1). Notably, certain combinations consistently predicted improvement (e.g., Age > 47, DIET = IECR or CER, showing TRUE 0.0 indicating 0 misclassifications in this sample), while others consistently predicted a lack of improvement (e.g., Cholesterol ≤ 3.9, FALSE 11.0). These patterns require validation in independent datasets before clinical implementation.

### 3.3. Logistic Regression Analysis: Independent Predictor Effects

**Figure 2:**
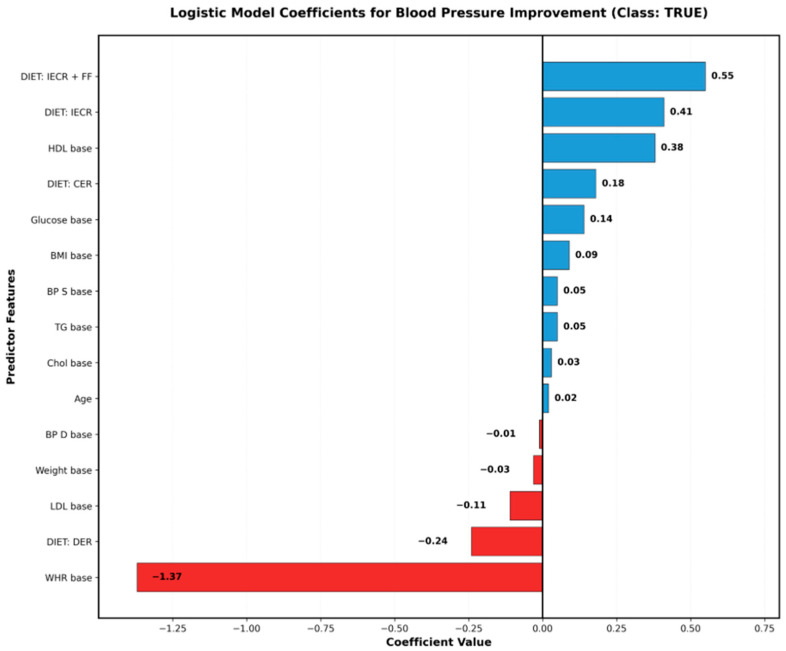
**Logistic Regression Coefficients from LMT Classifier for Blood Pressure Improvement.** Coefficient values represent the independent effect of each predictor on the log-odds of achieving ≥5 mmHg systolic blood pressure reduction. Positive coefficients (blue) indicate increased probability of improvement; negative coefficients (red) indicate decreased probability. WHR base = waist-to-hip ratio baseline; IECR = intermittent energy and carbohydrate restriction; FF = flexible fasting; CER = continuous energy restriction; DER = daily energy restriction.

These coefficients represent additive effects when other predictors are held. The logistic regression model embedded within the LMT classifier quantified the independent contribution of each predictor to blood pressure improvement probability (Figure 2). Unlike the decision tree’s hierarchical thresholds (Figure 1), these coefficients represent the additive effect of each feature when other predictors are held constant, assuming linear relationships on the logit scale constant.

#### 3.3.1. Dietary Intervention Effects

Dietary intervention type showed the strongest positive associations with blood pressure improvement (Figure 2):

- DIET: IECR + FF (coefficient = 0.55): The combined intermittent energy and carbohydrate restriction with flexible fasting showed the largest positive effect, increasing the log-odds of improvement by 0.55 units;
- DIET: IECR (coefficient = 0.41): Intermittent energy and carbohydrate restriction alone remained highly predictive;
- DIET: CER (coefficient = 0.18): Continuous energy restriction showed modest positive effects;
- DIET: DER (coefficient = −0.24): Daily energy restriction showed negative association with improvement, suggesting this approach may be less effective for blood pressure reduction in this specific population.

These findings indicate the hierarchical effectiveness of different dietary approaches in this cohort: IECR-based protocols (especially combined with flexible fasting) were associated with higher rates of blood pressure improvement compared to continuous daily restriction protocols.

#### 3.3.2. Metabolic Predictors

Among the baseline metabolic markers, HDL cholesterol showed the strongest positive association (coefficient = 0.38) (Figure 2), suggesting that individuals with better baseline lipid profiles may be more likely to achieve blood pressure improvements with dietary interventions in this population. This association may reflect underlying metabolic health that enhances responsiveness to lifestyle modification.

Glucose baseline showed a modest positive association (coefficient = 0.14), while LDL baseline showed negative association (coefficient = −0.11), indicating that higher LDL levels were associated with reduced likelihood of blood pressure response to dietary intervention in this sample, possibly reflecting greater baseline metabolic dysfunction or arterial stiffness.

#### 3.3.3. Anthropometric Predictors

Waist-to-hip ratio (WHR) emerged as the strongest negative predictor (coefficient = −1.27) (Figure 2), indicating that central adiposity was substantially associated with reduced probability of blood pressure improvement in this cohort. This large negative coefficient suggests that for every unit increase in WHR, the odds of achieving a ≥5 mmHg SBP reduction decreased by approximately 72% (OR = e^−1.27^ ≈ 0.28) in this sample, even when controlling for BMI and weight.

BMI baseline showed modest positive association (coefficient = 0.09), while the weight baseline showed a slight negative association (coefficient = −0.03) (Figure 2). The opposing directions of BMI and weight effects, combined with the strong negative WHR effect, suggest that body fat distribution (central vs. peripheral) may be more important than total adiposity for predicting blood pressure response, though this pattern requires confirmation in independent samples.

#### 3.3.4. Blood Pressure Baselines

Baseline systolic blood pressure showed minimal positive association (coefficient = 0.05), while diastolic blood pressure showed minimal negative association (coefficient = −0.01) (Figure 2). These small coefficients indicate that within the study range, absolute baseline blood pressure values had limited independent predictive value once other factors were considered, though the decision tree analysis (Figure 1) revealed important threshold effects (123 mmHg) that may not be captured by linear modeling.

#### 3.3.5. Age Effects

Age showed a minimal independent effect (coefficient = 0.02) in the logistic model (Figure 2), contrasting sharply with its prominent role in the decision tree analysis (Figure 1). This discrepancy indicates that age’s predictive value operates primarily through interactions with other features (particularly diet type) rather than as a linear main effect—highlighting the complementary insights provided by the two analytical approaches.

### 3.4. Comparison of Decision Tree and Logistic Regression Insights

The decision tree (Figure 1) and logistic regression (Figure 2) approaches revealed complementary patterns in this exploratory analysis:

#### 3.4.1. Convergent Findings

Both methods identified dietary intervention type as strongly associated with treatment success, with IECR and IECR + FF approaches showing superior outcomes compared to DER in this sample. Both methods also recognized associations between baseline metabolic markers (HDL, cholesterol, glucose) and treatment response, as well as the negative association of central adiposity with improvement.

#### 3.4.2. Divergent Insights

The decision tree emphasized age as a primary stratification feature with a specific threshold (47 years) for determining diet–response patterns (Figure 1), while the logistic model assigned age minimal independent weight (Figure 2). This difference reflects the decision tree’s ability to capture non-linear interactions: age appears to modify the effectiveness of different diets rather than having a constant linear effect across the population.

Similarly, baseline systolic blood pressure served as the primary decision tree split (threshold 123 mmHg) (Figure 1) but showed minimal coefficient in logistic regression (Figure 2), indicating threshold effects that linear models cannot capture. The decision tree revealed that individuals above this threshold may have fundamentally different response patterns in this cohort, while the logistic model assumes gradual linear changes in probability.

Conversely, WHR showed the strongest coefficient in logistic regression (coefficient = −1.27) (Figure 2) but did not appear in the main decision tree structure (Figure 1), suggesting that its effect may be relatively constant across patient subgroups rather than defining distinct categories of responders.

#### 3.4.3. Implications for Future Research

Together, these complementary analyses suggest potential patterns that warrant investigation in independent cohorts. The findings indicate that patient selection strategies may benefit from both categorical stratification (using age in years and baseline BP thresholds to guide diet type selection) and continuous risk assessment (using WHR, HDL, and other metabolic markers to estimate probability of success within assigned pathways). However, given the study’s limitation to premenopausal women without diagnosed hypertension and the absence of external validation, these patterns should be considered preliminary. The decision tree provides potential clinical algorithms (e.g., “For patients >47 years, consider IECR protocols”), while the logistic model quantifies how specific patient characteristics (high WHR, low HDL) may modify success probability within these pathways. Validation studies in broader populations—including men, postmenopausal women, and individuals with established hypertension—are essential before these findings can inform clinical practice.

## 4. Discussion

### 4.1. Principal Findings and Contribution to the Field

This study represents an exploratory application of machine learning approaches to identify baseline characteristics associated with blood pressure response to different intermittent fasting protocols in premenopausal women. Multiple classification algorithms (J48 decision tree, Logistic Model Tree, Random Forest) achieved comparable performance for the primary outcome of clinically significant systolic blood pressure reduction (≥5 mmHg), with AUC values of 0.79–0.80 and accuracy of 70–74%. The models successfully identified several factors associated with treatment response using readily available clinical parameters, though validation in independent cohorts is required before clinical implementation.

The complementary analytical approaches revealed distinct but convergent insights. Decision tree analysis identified a hierarchical stratification structure with baseline systolic blood pressure (threshold: 123 mmHg) as the primary decision point, followed by age in years-based stratification (threshold: 47 years) that determined which dietary interventions showed favorable outcomes. For participants over 47 years with elevated baseline blood pressure, IECR-based protocols (intermittent energy and carbohydrate restriction, particularly combined with flexible fasting) showed consistently favorable outcomes, while younger participants required more complex assessment incorporating cholesterol, BMI, and additional metabolic markers.

Logistic regression analysis, embedded within the LMT classifier, revealed that dietary intervention type exerted the strongest positive effects (IECR + FF coefficient = 0.55, IECR coefficient = 0.41), while waist-to-hip ratio emerged as the strongest negative predictor (coefficient = −1.27), indicating that central adiposity substantially reduced the probability of blood pressure improvement regardless of intervention type. Higher baseline HDL cholesterol showed positive associations (coefficient = 0.38), while the DER (daily energy restriction) protocol showed negative associations (coefficient = −0.24) in this cohort.

The divergence between decision tree and logistic regression findings provides methodological insights: age in years and baseline blood pressure operated primarily through threshold effects and interactions (prominent in decision trees) rather than linear main effects (minimal logistic regression coefficients), while WHR showed strong linear effects but did not define categorical responder groups. This complementarity suggests that optimal prediction may require both categorical stratification and continuous risk assessment.

However, several critical limitations constrain interpretation and generalizability. The study population consisted exclusively of premenopausal women without diagnosed hypertension, limiting applicability to men, postmenopausal women, and individuals with established hypertension. The absence of external validation means that identified thresholds and associations may represent sample-specific patterns rather than generalizable relationships. The modest sample size relative to predictors (EPV = 7.2) raises concerns about model stability, particularly for features appearing in deeper decision tree branches. These findings should be considered hypothesis-generating, requiring validation in diverse populations before informing clinical practice.

### 4.2. Positioning Within the Precision Nutrition Landscape

#### 4.2.1. Machine Learning in Nutritional Research

The application of machine learning to personalized nutrition has accelerated dramatically in recent years, with the field moving from conceptual frameworks to practical implementation [43,44]. Recent systematic reviews have documented the diverse applications of AI and machine learning in nutrition, including food image recognition, dietary intake assessment, nutrient composition analysis, and personalized diet recommendations [45,46]. However, most existing applications focus on dietary assessment or meal planning rather than predicting treatment response to specific nutritional interventions.

Our study contributes to an emerging subfield: using machine learning to identify treatment effect heterogeneity and predict individual response to dietary interventions [43,47]. This approach moves beyond population-level effect estimates toward identifying which individuals will benefit most from specific interventions—a central tenet of precision medicine. Recent work has demonstrated the feasibility of this approach across various nutritional contexts, including vitamin D supplementation response [48], weight loss intervention outcomes [49], and metabolic response to different macronutrient compositions [50]. However, application to intermittent fasting protocols specifically for blood pressure management represents a novel contribution.

#### 4.2.2. Interpretability and Clinical Implementation Potential

A critical distinction of our approach is the emphasis on model interpretability through the Logistic Model Tree architecture. While more complex “black box” models (deep neural networks, gradient boosting machines) might achieve marginally higher predictive accuracy, they sacrifice the transparency necessary for clinical adoption and mechanistic insight [51,52]. Recent position statements on AI in clinical nutrition have emphasized that interpretability is not merely a desirable feature but an ethical imperative, particularly in preventive healthcare contexts where patients must understand and engage with recommendations [53,54].

The hierarchical decision tree structure generated by the LMT classifier provides clinically intuitive decision pathways that mirror how clinicians naturally approach patient assessment: broad categorization followed by progressively refined evaluation [55]. This alignment with clinical reasoning facilitates integration into existing practice workflows, increasing the likelihood of real-world adoption. Recent surveys of healthcare providers indicate that interpretability ranks among the top factors influencing willingness to use AI-based clinical decision support tools [56].

However, the substantial limitations in population representativeness (premenopausal women only) and absence of external validation mean that clinical implementation remains premature. The identified decision rules and thresholds require confirmation in diverse cohorts before they can reliably inform clinical practice. Additionally, the model’s 26–30% misclassification rate, while comparable to other clinical prediction tools, necessitates careful consideration of how uncertain predictions would be handled in clinical decision-making.

### 4.3. Model Performance in Context

#### 4.3.1. Comparison with Existing Prediction Models

For the primary outcome (SBP ≥ 5 mmHg), the models achieved AUC values of 0.79–0.80 (LMT and Random Forest, respectively) with accuracy of 70–74%. These performance metrics compare favorably with existing clinical prediction models in related domains. Cardiovascular risk prediction models using traditional approaches (Framingham Risk Score, QRISK, SCORE) typically achieve AUCs between 0.70–0.78 for predicting cardiovascular events [57]. Machine learning models for predicting weight loss success with dietary interventions have reported AUCs ranging from 0.65–0.80, with most studies in the 0.70–0.75 range [58,59].

The sensitivity analyses revealed performance variation across outcome definitions (AUC range 0.73–0.80), with pulse pressure reduction ≥ 3 mmHg achieving the highest AUC (0.80) but systolic blood pressure reduction ≥ 5 mmHg selected as primary outcome due to superior clinical interpretability and guideline alignment. This variability indicates that different blood pressure components (systolic, diastolic, pulse pressure) are not equally predictable from the baseline characteristics assessed, though dietary intervention type and body composition remained important across all definitions.

Notably, a recent study using machine learning to predict metabolic biomarkers of intermittent fasting in mice achieved an F1-score of 0.88 using Random Forest with metabolomic data [60]. While this performance exceeds our results, direct comparison is complicated by differences in outcome definition (biomarker detection vs. clinical response), species (mice vs. humans), and data complexity (metabolomics vs. standard clinical parameters). The trade-off between model performance and data accessibility is critical: reliance on routine clinical measurements enhances potential applicability despite possibly limiting peak performance.

It is important to note that all performance estimates derive from internal validation (cross-validation) within the original dataset. External validation in independent cohorts may reveal substantially different performance, particularly if the training sample’s characteristics (premenopausal women, predominantly White European, recruited from specific UK trials) do not generalize to other populations.

#### 4.3.2. Interpretation of Predictive Accuracy

From a clinical perspective, accuracy of 70–74% means that approximately seven in ten patients would be correctly classified regarding the blood pressure improvement likelihood, while three in ten would be misclassified. This represents improvement over random classification (approximately 50% accuracy for balanced outcomes) but falls short of the certainty desired for clinical decision-making.

The AUC values of 0.79–0.80 indicate good discrimination between responders and non-responders, approaching the threshold (AUC ≥ 0.80) often considered excellent for clinical decision support [61]. However, AUC reflects rank-ordering ability (correctly ordering patients by improvement probability) rather than prediction accuracy for individual patients. A model with AUC 0.80 can still misclassify many individuals, particularly those with intermediate predicted probabilities.

### 4.4. Baseline Blood Pressure Threshold and Patient Stratification

#### 4.4.1. The 123 mmHg Threshold in Decision Tree Analysis

The decision tree identified baseline systolic blood pressure of 123 mmHg as the primary splitting feature, with patients above this threshold showing a higher probability of achieving ≥5 mmHg reduction. This threshold falls within the elevated blood pressure range (120–129 mmHg systolic) defined by current guidelines [4], marking the transition between normal and early Stage 1 Hypertensive (HTN) states.

However, several factors complicate the interpretation of this threshold’s clinical significance. First, the threshold of 123 mmHg may represent a sample-specific pattern rather than a universal physiological transition point. With a modest sample size (*n* = 222) and multiple potential splitting features, decision trees can identify thresholds that maximize classification in the training data but do not generalize to independent populations. The fact that baseline systolic blood pressure showed minimal independent effect in logistic regression analysis (coefficient = 0.05) suggests that this feature’s predictive value operates primarily through interactions or threshold effects rather than continuous linear relationships.

Second, the finding that patients with baseline SBP > 123 mmHg showed higher improvement rates may partially reflect regression to the mean—a statistical phenomenon where extreme values tend to move toward the population average on repeated measurement. Individuals with higher baseline blood pressure have more room for absolute reduction and may show larger changes even without true intervention effects. This interpretation is supported by the fact that the outcome was defined as absolute reduction (≥5 mmHg) rather than percent change or adjustment to baseline.

Third, the biological mechanisms underlying any true threshold effect at 123 mmHg could not be determined from these data. While one might speculate about vascular remodeling, endothelial dysfunction, or metabolic changes occurring at this blood pressure level, the current study included no measurements of these putative mechanisms. Future mechanistic studies incorporating vascular function assessment, inflammatory markers, or metabolic profiling would be needed to elucidate whether physiological transitions occur at this threshold.

#### 4.4.2. Differential Complexity in Patient Assessment

The decision tree revealed asymmetric complexity in prediction pathways: patients with baseline SBP > 123 mmHg were primarily stratified by age in years and dietary intervention type, while those with SBP ≤ 123 mmHg required more complex assessment incorporating cholesterol, BMI, triglycerides, and other metabolic markers. This pattern suggests that baseline blood pressure level may influence which other characteristics become important for prediction.

Several non-mutually exclusive explanations could account for this observation:

Statistical Explanation: The sample contained more individuals with SBP > 123 mmHg who improved (creating clearer signal for classification), while the SBP ≤ 123 mmHg group had more heterogeneous outcomes requiring multiple features to achieve acceptable classification. This could reflect true biological heterogeneity or simply sampling variability.

Ceiling Effects: Individuals with lower baseline blood pressure have limited capacity for further reduction before reaching hypotensive levels, creating a more restricted outcome range that depends on subtle individual differences. Conversely, those with higher baseline pressure have greater reduction potential, making intervention type the dominant factor.

Different Response Mechanisms: It is possible (though not demonstrated by these data) that blood pressure reduction mechanisms differ between normal BP/elevated BP and stage 1 hypertension (HTN) individuals. However, without mechanistic measurements, this remains speculative.

Unmeasured Confounding: The differential stratification patterns could reflect features not included in the model that correlate with baseline blood pressure—such as dietary sodium intake, physical activity, stress levels, or medication use—that influence response differently across blood pressure ranges.

### 4.5. Complementary Insights from Decision Tree and Logistic Regression Analyses

The use of both decision tree and logistic regression approaches provided complementary insights that neither method alone could capture. Decision trees excel at identifying threshold effects and interactions—revealing, for example, that age 47 years and baseline SBP 123 mmHg represent critical decision points where optimal intervention strategies change. These categorical thresholds align with clinical decision-making patterns and provide actionable rules.

Conversely, logistic regression quantifies independent linear effects, revealing that WHR, dietary intervention type, and HDL exert continuous influences on improvement probability. The fact that age in years showed a minimal independent effect in logistic regression (coefficient = 0.02), despite being a primary decision tree split, illustrates a key distinction: age in years’ predictive value operates through interactions with other features (particularly diet type) rather than as a main effect.

This methodological complementarity has implications for clinical implementation. Decision tree thresholds suggest categorical patient stratification (e.g., ‘For patients >47 years with baseline SBP > 123, use IECR protocols’), while logistic coefficients enable continuous probability estimation (e.g., ‘Each 0.1 unit increase in WHR reduces improvement probability by X%’). Optimal clinical decision support might incorporate both approaches—using decision rules for initial stratification and continuous risk scores for nuanced probability assessment within strata.

However, the divergence between methods also highlights model uncertainty. When decision tree and logistic regression disagree on feature importance (as with age in years and WHR), this suggests that the underlying relationships are complex and may not be fully captured by either modeling approach. External validation will be critical to determine which patterns represent robust, generalizable relationships versus sample-specific artifacts.

### 4.6. Considerations for Future Clinical Implementation

#### 4.6.1. Data Requirements and Accessibility

A potential strength of this modeling approach is its reliance on routinely collected clinical parameters. The required input features—age in years, waist and hip circumference for WHR calculation, blood pressure, and standard lipid and glucose panels—are typically available in primary care and nutrition counseling settings, requiring no specialized testing or additional patient burden beyond standard assessment. This accessibility contrasts with precision nutrition approaches requiring genomic sequencing, metabolomic profiling, or continuous glucose monitoring, which may be cost-prohibitive or logistically challenging in many settings [54].

However, the feasibility of collecting these parameters should not be conflated with readiness for clinical deployment. Even with accessible data, multiple barriers to implementation must be addressed before clinical use would be appropriate.

#### 4.6.2. Implementation Challenges Beyond Validation

Even after successful validation, implementation would face additional challenges:

Integration with Clinical Workflows: Seamless integration with electronic health records and clinical decision support systems requires technical infrastructure that varies widely across healthcare settings. Recent systematic reviews of AI implementation in clinical nutrition have identified key requirements including user-friendly interfaces, integration with existing systems, clear presentation of uncertainty, and mechanisms for clinician override [53,54].

### 4.7. Limitations and Interpretation Caveats

#### 4.7.1. Sample Characteristics and Generalizability

The study population consisted exclusively of premenopausal women (ages 30–45 years) who were overweight or obese, predominantly White European, and recruited through specific UK-based trials. This homogeneity substantially limits generalizability across several dimensions:

Sex and Hormonal Status: The exclusion of men means that all identified predictive patterns—including the age in years threshold at 47 years, the dietary intervention effects, and the strong negative association of WHR with improvement—remain untested in males. Sex differences in body composition, metabolic regulation, hormonal influences on appetite and metabolism, and cardiovascular physiology could substantially alter which factors predict blood pressure response to IF [6,62]. Similarly, the restriction to premenopausal women precludes an assessment of whether postmenopausal women show similar patterns. Given that the identified age in years threshold (47 years) approaches typical perimenopause onset, and that estrogen status affects vascular function, metabolic health, and body fat distribution, the predictive model may not apply to postmenopausal women [63].

Ethnic and Genetic Diversity: The predominantly White European cohort limits applicability to other ethnic populations who may have different genetic predispositions, metabolic baselines, body composition patterns, and cultural dietary practices. Research has documented ethnic differences in obesity phenotypes (particularly visceral adiposity distribution), insulin resistance patterns, and cardiovascular risk profiles that could substantially influence IF response [64,65]. For example, the strong negative association of WHR with blood pressure improvement observed in this study might differ in populations with different body fat distribution patterns.

Age Range: The narrow age range (30–45 years) excludes older adults who represent a substantial proportion of individuals with elevated blood pressure requiring intervention. The identified age threshold of 47 years, which distinguished patients requiring different dietary approaches in the decision tree analysis, cannot be validated or contextualized within the study sample since no participants exceeded age 45 years. Aging-related changes in vascular compliance, metabolic rate, body composition, medication use, and comorbidity burden could alter the relative importance of different predictive factors [66,67]. Whether the age-stratified dietary recommendations identified here apply to older adults remains entirely unknown.

Baseline Health Status: Participants were free of diagnosed cardiovascular disease, diabetes, and hypertension, representing a relatively healthy population despite being overweight or obese. Individuals with established disease may exhibit fundamentally different response patterns due to:

- Antihypertensive medication use (not present in this sample).
- Other medications affecting metabolism or blood pressure disease-related metabolic alterations (advanced insulin resistance, diabetic complications).
- Vascular damage or remodeling reducing responsiveness to lifestyle intervention.
- Different baseline blood pressure ranges and reduction potential.

The decision tree’s primary split at baseline SBP 123 mmHg stratified individuals within the normal-to-elevated range; whether similar thresholds apply to individuals with Stage 1 or Stage 2 hypertension is unknown.

Sample Size and Statistical Power: With 222 participants and 13 predictors, the events-per-feature ratio (EPV = 7.2 for the minority class) fell below the ideal threshold of 10, raising concerns about model stability. Features appearing in deeper decision tree branches are based on progressively smaller subgroups, increasing the risk that identified thresholds represent sample-specific noise rather than generalizable patterns. The logistic regression coefficients, while more stable than tree-based methods, still carry substantial uncertainty given the modest sample size.

Future validation studies should deliberately include diverse populations across sex, age, ethnicity, and health status to assess model transportability. It is likely that population-specific recalibration or even entirely different models will be needed for different demographic and clinical groups.

#### 4.7.2. Outcome Definition and Measurement

The selection of a systolic blood pressure reduction ≥ 5 mmHg as the primary outcome, while clinically relevant and guideline-aligned, represents one of multiple valid approaches to defining blood pressure improvement. The sensitivity analyses revealed that model performance, and potentially the relative importance of predictors, varies across outcome definitions:

Alternative Blood Pressure Metrics:

Different blood pressure components capture distinct physiological aspects. Systolic pressure reflects arterial stiffness and pulsatile load, diastolic pressure indicates peripheral resistance, pulse pressure represents arterial compliance, and mean arterial pressure provides a flow-weighted average relevant to organ perfusion [68,69]. The fact that a pulse pressure reduction ≥ 3 mmHg achieved marginally higher discrimination (AUC 0.80) than systolic reduction ≥ 5 mmHg (AUC 0.79–0.80) suggests that arterial compliance changes may be slightly more predictable from baseline characteristics than absolute systolic changes. However, whether the same predictors would emerge as important for different outcomes (e.g., mean arterial pressure, diastolic pressure) was not systematically evaluated.

Pulse pressure was evaluated as a secondary and sensitivity outcome in this study; however, its clinical relevance in younger, premenopausal, and largely normotensive populations is limited. In such groups, pulse pressure primarily reflects short-term hemodynamic variability rather than structural arterial stiffness, which is more characteristic of older or atherosclerotic populations.

Accordingly, any associations identified with pulse pressure outcomes should be interpreted cautiously and should not be extrapolated to cardiovascular risk reduction or long-term clinical benefit. This limitation further supports the prioritization of systolic blood pressure reduction as the primary outcome in the present analysis, given its clearer clinical relevance and stronger guideline alignment in this population.

Threshold Effects:

Model performance varied across improvement thresholds (any improvement > 0 mmHg: AUC 0.78; ≥3 mmHg: AUC 0.78; ≥5 mmHg: AUC 0.79–0.80), indicating that factors predicting any blood pressure reduction may partially differ from those predicting clinically meaningful reductions. The 5 mmHg threshold was selected for clinical relevance, but other thresholds might be more appropriate for different clinical contexts.

Measurement Method: All blood pressure measurements were clinic-based, subject to white-coat effects, measurement error, and lack of information about diurnal variation. Ambulatory or home blood pressure monitoring would provide more comprehensive assessment and might reveal different predictive patterns, particularly if some interventions affect daytime versus nighttime blood pressure differentially [70].

Binary vs. Continuous Outcomes: The binary classification (improved/not improved) is clinically pragmatic but discards information about the magnitude of change. A patient with a 4 mmHg reduction is classified identically to one with no change, while differing only slightly from a patient with a 6 mmHg reduction. Continuous modeling of blood pressure change magnitude might reveal different predictive patterns but would require larger sample sizes and different analytical approaches.

Short-term vs. Long-term Outcomes: These limitations are compounded by the focus on short-term outcomes (see Section 4.7.3).

#### 4.7.3. Intervention Duration and Long-Term Sustainability

The analysis focused exclusively on 12-week outcomes, representing short-term response to IF interventions. This timeframe is insufficient for several critical questions:

Sustained Blood Pressure Benefits: Blood pressure management requires long-term maintenance, and initial responses may not predict sustained benefits. Meta-analytic evidence suggests that blood pressure reductions with lifestyle interventions may attenuate over time, potentially due to decreased adherence, metabolic adaptation, or partial weight regain [71,72]. Factors predicting which individuals maintain blood pressure improvements beyond 12 weeks may differ entirely from those predicting initial response.

Adherence and Dropout: Recent research has documented substantial dropout rates with IF interventions (10–40% across studies), with adherence declining markedly after the initial months [7,8,9,22,25]. The current models predict physiological response among those who complete 12 weeks of intervention but do not predict adherence, dropout, or long-term sustainability. A clinically useful prediction tool might need to predict not “who will respond if they adhere” but rather “who will both adhere and respond”—requiring incorporation of behavioral, psychological, and lifestyle features beyond the clinical parameters used here.

Weight Regain and Blood Pressure Rebound: If blood pressure improvements depend primarily on weight loss, individuals who regain weight may experience blood pressure rebound regardless of initial response. The current models include baseline weight and BMI but do not capture factors predicting weight maintenance.

Model Relevance for Chronic Management: Even if the identified predictors (WHR, dietary intervention type, age in years, baseline blood pressure, HDL) accurately predict 12-week outcomes, their relevance for 1-year, 5-year, or lifelong blood pressure management remains unknown. Different factors might predict long-term success, necessitating the development of separate models for different time horizons.

Future research should extend follow-up to at least 6–12 months, ideally longer, and develop models predicting sustained benefit rather than just initial response.

#### 4.7.4. Unmeasured Confounding and Missing Predictors

The models relied exclusively on readily available clinical parameters (demographics, anthropometrics, blood pressure, standard lipid and glucose panels), while numerous potentially important predictors remained unmeasured. Important biological markers were absent, including renin–angiotensin–aldosterone system activity (plasma renin, aldosterone levels), electrolytes (sodium, potassium), inflammatory markers (hsCRP, IL-6), direct insulin resistance measures (HOMA-IR, fasting insulin), and vascular function assessments. Additionally, dietary intake patterns, physical activity levels, sleep quality, medication use (NSAIDs, oral contraceptives), and behavioral features (self-efficacy, motivation, adherence history) were not captured. The absence of these features means that observed associations may partially reflect unmeasured confounding, and model performance might improve with their inclusion. Future validation studies should prioritize adding RAAS markers, electrolytes, inflammatory markers, and insulin resistance assessment to enhance both prediction and mechanistic understanding.

#### 4.7.5. Model Stability, EPV, and Overfitting Risk

Although the overall sample size and global events-per-feature (EPV) ratio were acceptable for exploratory prediction modeling, the hierarchical structure of the Logistic Model Tree introduces an important additional consideration: the effective EPV within individual terminal nodes. As the tree grows deeper, observations are partitioned into increasingly smaller subgroups, and logistic regression models fitted at terminal nodes may rely on a limited number of events.

This reduction in node-level EPV increases the risk of coefficient instability, exaggerated effect sizes, and sample-specific decision thresholds. Consequently, splits appearing in deeper branches—particularly those involving metabolic or lipid thresholds—should be interpreted with caution and should not be assumed to represent true biological discontinuities.

While 10-fold cross-validation was employed to reduce overfitting and assess internal stability, cross-validation cannot fully compensate for sparse data within terminal nodes. The LMT framework in this study is therefore best viewed as an exploratory method for identifying candidate interactions and stratification patterns, rather than as a definitive or deployable clinical prediction model.

#### 4.7.6. Clinical Interpretation, Model Limitations, and Implications for Future Research

The present findings should not be interpreted as evidence supporting clinical decision-making or the individualized prescription of intermittent fasting protocols. The models were developed using secondary data from relatively small and demographically homogeneous cohorts of premenopausal women without diagnosed hypertension and were internally—but not externally—validated, which limits their generalizability.

Accordingly, the analytical framework should be regarded as exploratory and hypothesis-generating. The identified associations and interaction patterns involving age in years, dietary intervention type, central adiposity, baseline blood pressure, and metabolic markers are best viewed as candidate signals for further investigation rather than as stable or clinically actionable thresholds.

External validation in independent, larger, and more diverse populations—including men, postmenopausal women, and individuals with established hypertension—is required to determine the robustness and potential clinical relevance of these findings. Future studies should prioritize prospective designs and validated models before any consideration of clinical application.

### 4.8. Clinical and Public Health Implications

#### 4.8.1. Preliminary Insights for Precision Nutrition

This exploratory analysis suggests that machine learning approaches using readily available clinical parameters can identify potential patterns of differential response to intermittent fasting protocols for blood pressure management. Specifically, the findings indicate that:

- Dietary intervention type (IECR-based protocols vs. continuous daily restriction) may be an important determinant of blood pressure response;
- Central adiposity (measured by WHR) may substantially influence the probability of improvement;
- Age-based stratification might inform dietary protocol selection, though the identified 47-year threshold requires validation;
- Baseline systolic blood pressure level may determine which additional factors become important for prediction.

If these patterns prove robust in external validation across diverse populations, they could potentially inform more personalized dietary intervention strategies. However, the substantial limitations discussed above—particularly the restriction to premenopausal women, absence of external validation, and modest sample size—mean that clinical implementation remains premature.

#### 4.8.2. Analytical Insights for Precision Nutrition

Beyond the specific findings about blood pressure and intermittent fasting, this study demonstrates the value of combining multiple analytical approaches to capture different aspects of predictor–outcome relationships:

Complementary Methods: Decision trees revealed threshold effects and interactions (age 47 years, baseline SBP 123 mmHg) that logistic regression, assuming linearity, could not capture. Conversely, logistic regression quantified continuous effects (WHR, dietary protocols) and provided stable coefficient estimates. Using both methods provided a more complete picture than either alone.

Interpretability Emphasis: By prioritizing interpretable models over maximum predictive accuracy, the analysis generated clinically actionable decision rules (if validated) and insights into which patient characteristics matter for prediction. This transparency facilitates clinical adoption, mechanistic investigation, and patient understanding.

Multiple Outcome Definitions: Systematic evaluation of seven different blood pressure outcome definitions revealed that predictive patterns are relatively robust but performance varies, providing guidance for future research on optimal outcome selection.

These methodological insights may inform future precision nutrition research even if the specific findings on intermittent fasting and blood pressure do not generalize.

### 4.9. Study Limitations and Future Validation of Decision Thresholds

Future external validation studies should incorporate systematic threshold sensitivity analysis to assess whether deeper-branch thresholds replicate or whether alternative cutoffs perform similarly, thereby evaluating the stability of decision splits identified in our tree. This is particularly crucial for thresholds appearing in deeper branches where sample sizes are smallest. Such analyses should:

(1) Test whether the exact numerical cutoffs identified (e.g., cholesterol ≤ 3.9 mmol/L, BMI > 48.1 kg/m^2^, triglycerides ≤ 2.3 mmol/L) replicate in independent cohorts.
(2) Evaluate whether alternative thresholds within clinically plausible ranges (e.g., ±10–20% of identified values) demonstrate equivalent predictive performance.
(3) Assess whether simpler categorizations based on clinical guidelines or distribution-based cutoffs (e.g., median splits, tertiles) perform comparably to the specific thresholds identified in our training sample.

Thresholds that prove robust across these sensitivity analyses likely represent genuine biological inflection points in treatment response, whereas those sensitive to minor perturbations should be interpreted as exploratory findings requiring validation and potential refinement before clinical application.

## 5. Conclusions

This study demonstrates the application of machine learning approaches to identify factors associated with blood pressure response to intermittent fasting interventions in premenopausal women. Using multiple analytical methods—decision tree classifiers, Logistic Model Trees, and Random Forest algorithms—the models achieved an accuracy ranging from 70–77% and AUC values of 0.78–0.80 in distinguishing participants who achieved clinically significant systolic blood pressure reduction (≥5 mmHg) from those who did not.

The complementary analytical approaches revealed several key patterns in this cohort. Dietary intervention type emerged as the strongest predictor across both decision tree and logistic regression analyses, with IECR-based protocols (particularly IECR + FF) showing superior associations with blood pressure improvement compared to continuous daily energy restriction. Age-based stratification proved critical in the decision tree analysis, with distinct response patterns observed for participants above and below 47 years, particularly in determining which dietary protocols were most effective. Waist-to-hip ratio emerged as the strongest negative predictor in logistic regression analysis, suggesting that central adiposity may substantially reduce the likelihood of blood pressure improvement regardless of intervention type.

The decision tree analysis identified specific thresholds that may inform patient stratification: baseline systolic blood pressure of 123 mmHg served as the primary decision point, with age (47 years) and various metabolic markers (cholesterol 3.9 mmol/L, BMI 39.1 kg/m^2^, triglycerides 2.3 mmol/L) providing additional stratification in specific subgroups. These threshold-based decision rules offer potential clinical algorithms, though their utility requires validation in independent populations.

The divergence between decision tree and logistic regression findings provides important methodological insights. While the decision tree emphasized age in years and baseline blood pressure as primary stratification features through threshold effects, the logistic regression model identified WHR, dietary intervention type, and HDL as the strongest continuous predictors. This complementarity suggests that optimal prediction may require both categorical patient stratification (identifying which diet type to consider based on age in years and baseline characteristics) and continuous risk assessment (quantifying probability of success based on body composition and metabolic markers).

However, several important limitations constrain the generalizability and clinical applicability of these findings. First, the study population consisted exclusively of premenopausal women without diagnosed hypertension, limiting applicability to men, postmenopausal women, and individuals with established hypertension—populations that represent the majority of candidates for blood pressure-lowering interventions. Second, the absence of external validation means that model performance in independent datasets remains unknown, and the identified thresholds and decision rules may reflect sample-specific patterns rather than generalizable relationships. Third, the modest sample size relative to the number of predictors (EPV = 7.2 for the minority class) suggests that some predictor coefficients may be unstable, particularly for features appearing in deeper branches of the decision tree.

The finding that pulse pressure reduction ≥ 3 mmHg achieved slightly higher AUC (0.80) than systolic blood pressure reduction ≥ 5 mmHg raises questions about whether arterial compliance improvements may be more predictable than absolute blood pressure changes in this population. This warrants further investigation, particularly given that pulse pressure is an established marker of arterial stiffness and cardiovascular risk in older adults.

Future research priorities include: (1) external validation in independent cohorts, particularly in men, postmenopausal women, and individuals with diagnosed hypertension; (2) prospective studies comparing outcomes when dietary protocols are selected based on these predictive models versus standard care; (3) investigation of the biological mechanisms underlying the observed age–diet interactions and the strong negative association between central adiposity and treatment response; (4) evaluation of whether the identified thresholds (age 47 years, baseline SBP 123 mmHg) represent true physiological transitions or sample-specific artifacts; and (5) assessment of whether incorporating additional biomarkers (inflammatory markers, insulin resistance indices, genetic variants) or digital health data (sleep patterns, physical activity, dietary adherence) improves predictive accuracy beyond the readily available clinical parameters.

If validated in broader populations, this framework could potentially inform personalized dietary intervention strategies by matching patients to specific IF protocols based on age in years, baseline metabolic profile, and body composition. The use of readily available clinical parameters (age in years, blood pressure, lipid profiles, anthropometric measurements) without requiring specialized testing enhances potential implementability. However, the substantial limitations in population representativeness and absence of external validation mean that clinical implementation remains premature. These findings should be considered hypothesis-generating, providing preliminary evidence that personalized selection of IF protocols based on patient phenotype may be feasible, but requiring rigorous validation before influencing clinical practice.

This study represents an exploratory step toward understanding which patient characteristics may predict blood pressure response to different intermittent fasting protocols. While the identified patterns are intriguing and the analytical approach demonstrates the value of combining multiple machine learning methods to capture both threshold effects and continuous relationships, translation to clinical decision-making awaits confirmation in diverse populations and prospective validation studies.

## Figures and Tables

**Table 1:** Average and STD of Features.

Feature	Average	STDEV
Age	44.0 (years)	7
Weight	83.0 (kg)	16
Waist	103.0 (cm)	14
Hip	111.0 (cm)	11
Waist–Hip Ratio	0.9	0.1
BMI	31.0 (kg/m^2^)	5
D-BP	75.0 (mmHg)	11
S-BP	121.0 (mmHg)	17
Cholesterol	5.3 (mmol/L)	1.0
LDL	3.3 (mmol/L)	0.9
HDL	1.5 (mmol/L)	0.4
Glucose	4.9 (mmol/L)	0.4
Triglyceride	1.2 (mmol/L)	0.6

**Table 2:** Diet regimes.

Intervention Name	Details	Reference
CER	Continuous Energy Restriction. 7-day-a-week trial; eating restricted calories every day.	Harvie et al. 2011 [34]
IER	Intermittent Energy Restriction. 2-day-a-week trial; eating restricted calories only two days a week.	Harvie et al. 2011 [34]
IECR	Intermittent Energy and Carbohydrate Restriction; eating restricted calories only two days a week.	Harvie et al. 2013 [35]
IECR + PF	Intermittent Energy and Carbohydrate Restriction + free Protein and Fat; eating restricted calories only two days a week.	Harvie et al. 2013 [35]
DER	Daily Energy Restriction; eating restricted calories every day.	Harvie et al. 2013 [35]

**Table 3:** Accuracy and AUC Performance of Three Classification Algorithms for Predicting Blood Pressure Improvement Using Different Outcome Thresholds.

Measure Threshold		Classifier	Accuracy	AUC
	Pulse Pressure	J48	73%	0.65
Pulse Pressure (SBP-DBP) > 0 mmHg		LMT	75%	0.76
		Random Forest	73%	0.76
	Pulse Pressure	J48	65%	0.62
Pulse Pressure (SBP-DBP) ≥ 3 mmHg		LMT	76%	0.80
		Random Forest	67%	0.73
	Pulse Pressure	J48	66%	0.62
Pulse Pressure (SBP-DBP) ≥ 5 mmHg		LMT	71%	0.79
		Random Forest	67%	0.71
	Pulse Pressure	J48	69%	0.68
SBP ≥ 5 mmHg		LMT	70%	0.79
		Random Forest	74%	0.80
	Pulse Pressure	J48	68%	0.66
SBP ≥ 3 mmHg		LMT	73%	0.78
		Random Forest	71%	0.76
	Pulse Pressure	J48	62%	0.52
SBP > 0 mmHg		LMT	77%	0.78
		Random Forest	70%	0.73
	Pulse Pressure	J48	62%	0.61
DBP ≥ 3 mmHg		LMT	73%	0.73
		Random Forest	68%	0.69
	Pulse Pressure	J48	74%	0.65
SBP ≥ 5 mmHg AND DBP ≥ 3 mmHg		LMT	73%	0.74
		Random Forest	73%	0.76
	Pulse Pressure	J48	65%	0.63
SBP ≥ 5 mmHg OR DBP ≥ 3 mmHg		LMT	73%	0.74
		Random Forest	73%	0.76

## Data Availability

The table containing the data of this research may be found at the following link: https://github.com/shulash/Blood-Pressure-and-Intermittent-Fasting/issues/1 (accessed on 12 January 2026). Additional data will be provided upon request to shulash@openu.ac.il.

## Abbreviations

The following abbreviations are used in this manuscript:

IF	Intermittent Fasting
S-BP	Systolic Blood Pressure
D-BP	Diastolic Blood Pressure
MAP	Mean Arterial Pressure
LMT	Logistic Model Tree
AUC	Area Under Curve
